# Borax

**Published:** 1894-12

**Authors:** 


It may not be generally known, but is nevertheless, a fact that borax will precipitate morphine from alkaline or slightly acid solution. The reaction has frequently been utilized by the writer in the assay of opium, etc. The precipitate is purer of a white color than it is when ammonia is the precipitant while the yield of crystals is fully as large.

